# A comprehensive study on freshwater pufferfish *Pao baileyi*: profiles of toxin possession, accumulation, and uptake

**DOI:** 10.3389/fvets.2026.1899689

**Published:** 2026-07-27

**Authors:** Hongchen Zhu

**Affiliations:** 1 State Key Laboratory of Food Science and Resources, Nanchang University, Nanchang, Jiangxi, China; 2 International Institute of Food Innovation Co., Ltd., Nanchang University, Nanchang, Jiangxi, China; 3 School of Food Science and Technology, Nanchang University, Nanchang, Jiangxi, China

**Keywords:** Intrarectal administration, *Pao baileyi*, pufferfish, saxitoxin (STX), tetrodotoxin (TTX), tissue slice incubation

## Abstract

Freshwater pufferfish, primarily belonging to the genus *Pao*, constitute an important branch in the family Tetraodontidae. Toxic freshwater pufferfish contain saxitoxin (STX) but lack tetrodotoxin (TTX), which is commonly found in marine pufferfish. Here, we focused on the freshwater pufferfish *Pao baileyi* and assessed the toxicity and toxin distribution patterns in wild individuals. Subsequently, we conducted intrarectal toxin administration *in vivo* and tissue slice incubation *in vitro* using artificially bred nontoxic individuals. Toxin analyses showed that wild *P. baileyi* possessed only STX, with relatively high STX concentrations in the intestine and muscle. In the intrarectal toxin administration, nontoxic individuals acquired a dose of 5 nmol STX/individual and the STX amount in the intestine, liver, muscle, and skin was quantified at 24, 48, and 72 h. At 24 and 72 h, the sum of relative STX amount across all tissues investigated was 59.52 and 56.49%, respectively, obviously higher than that observed at 48 h (21.37%). In the tissue slice incubation, slices of these four tissues prepared from the same batch of nontoxic *P. baileyi*, were incubated in solutions containing TTX and STX and toxin taken up in the tissue was quantified over time. The dynamic changes of STX within the liver and skin over the 24-to 72 -h period are similar between *in vivo* and *in vitro* experiments, however, those of STX in the intestine totally differ. Tissues of *P. baileyi* could selectively take up STX. This study reveals the profiles of toxin possession, accumulation, and uptake of representative *Pao* freshwater pufferfish.

## 1 Introduction

Pufferfish of the family Tetraodontidae are widely distributed in coastal waters of Japan, inland and coastal regions of China, the Mekong River basin, the Amazon rainforest, and the Indo-Pacific (1–4). They are typically classified into two categories based on their habitat: marine and freshwater pufferfish. Marine pufferfish of the genus *Takifugu* inhabiting Japan coastal waters contain tetrodotoxin (TTX) in high concentrations within their liver, ovaries, and skin, which is the primary toxic component in the body (5–9). TTX is one of the most potent neurotoxins discovered to date and was once considered the most toxic nonprotein toxin in nature (10). It causes systemic paralysis, respiratory failure, and even death by blocking voltage-gated sodium channels on membranes of neuron cells and skeletal muscle fibers (11–14). Consequently, in East Asian regions like Japan and China, where consumption of pufferfish is a prevalent culinary tradition, cases of food poisoning from ingesting toxic marine pufferfish frequently occur (15, 16). Although the exact origin of TTX in these marine pufferfish remains unknown, it is widely speculated that TTX accumulates through the food chain, beginning with TTX-producing bacteria (8, 17–19). When separated from their natural food chains and fed artificial diets, marine pufferfish could turn from toxic to nontoxic (20).

On the other hand, freshwater pufferfish represented by the genera *Pao* and *Leidon*, inhabiting freshwater areas such as Southeast Asia, South Asia, and the Amazon rainforest, are also highly toxic (21). Generally, these freshwater pufferfish do not contain TTX but instead bear high levels of saxitoxins (STXs), including saxitoxin (STX), neosaxitoxin (neoSTX), and decarbamoylsaxitoxin (dcSTX), mainly in the skin and ovaries (22–24). STXs are a group of highly toxic neurotoxins belonging to the guanidine-containing alkaloid family, and same as TTX they are also sodium channel blockers that inhibit the conduction of action potentials by selectively binding to the sodium ion permeation pathway of voltage-gated sodium channels and blocking Na^+^ access to the selectivity filter from the extracellular side of the plasma membrane in excitable cells (25, 26). Therefore, the symptoms of poisoning in humans after accidentally consuming toxic freshwater pufferfish resemble those caused by TTX poisoning: mild cases may result in localized paralysis, while severe cases could lead to life-threatening respiratory distress. Since wild freshwater pufferfish consumption is customary in Southeast Asian countries such as Cambodia, Thailand, and Laos, and the public lacks knowledge about toxic pufferfish species and their toxicity characteristics, fatalities from accidental ingestion of toxic freshwater pufferfish occur periodically (27). STXs accumulated in freshwater pufferfish are presumed to be exogenous through the food chain originating from STX-producing dinoflagellates in marine environments and STX-producing cyanobacteria in freshwater environments (28–31). Research on freshwater pufferfish is still in its early stages, and information regarding their toxicity characteristics, toxin kinetics, and toxin accumulation capacity remains scarce.

Distinct from freshwater pufferfish, which exclusively possess STXs, in addition to the aforementioned genus *Takifugu* primarily bearing TTX, there are some other genera of marine pufferfish, such as the genus *Sphoeroides* from Florida, *Arothron* from the Philippines and Japanese coastal waters, and *Canthigaster* inhabiting the Caribbean Sea and Japanese subtropical waters, concurrently having TTX and STXs (22, 28, 30, 32, 33). Despite dedicating considerable time and effort over the years to exploring the possession profiles of TTX and STXs in wild pufferfish across different genera and species within the family Tetraodontidae and summarizing the types and concentrations of toxins present in various species including *Takifugu* and *Pao*, the understanding of dynamic changes and metabolic pathways of toxins in pufferfish is limited. Thus, research on the toxin accumulation capacity at the individual level and the toxin uptake capacity at the tissue level in pufferfish has become particularly crucial. Benefiting from the finding that pufferfish artificially reared from hatching with nontoxic feed do not contain TTX/STXs, nontoxic artificial rearing of various pufferfish has become feasible. Consequently, research utilizing these artificially reared pufferfish has gradually gained momentum. Several *in vivo* toxin administration experiments have been conducted on these artificially reared nontoxic pufferfish (34–39). In TTX administration on the *Takifugu* marine pufferfish, TTX kinetics were observed: TTX was transferred from the intestine to the liver, and subsequently from the liver to either the skin or ovaries, which may differ between sexes and change as the individual grows and/or matures (38, 40, 41). After intrarectal administration of TTX and STX to the euryhaline freshwater pufferfish *Dichotomyctere fluviatilis*, selective accumulation of these toxins in this species was observed (42). A significant proportion of STX was retained by *D. fluviatilis*, but ultimately less than 0.1% of TTX accumulated in the body, revealing that this species selectively accumulates STX. However, since only the intestine and liver showed detectable traces of the toxins, it failed to fully elucidate the kinetic and dynamic profiles of toxins across the tissues of freshwater pufferfish.

The *in vitro* tissue slice incubation technique utilizing pufferfish has become mature. Studies by Nagashima et al. (43) and Matsumoto et al. (44) using this technique demonstrated that, different from nontoxic common marine fish such as parrot-bass *Oplegnathus fasciatus* and green ling *Hexagrammos otakii*, the liver of *Takifugu* marine pufferfish could take up TTX, while showing almost no STXs uptake ability, similar to nontoxic common marine fish. Zhu et al. (42) applied the method developed by Nagashima et al. (43) to multiple tissues of *Takifugu rubripes* and *D. fluviatilis*, and found that unlike *T. rubripes*, whose intestine, liver, and skin selectively took up TTX, the three tissues of *D. fluviatilis* selectively took up STXs, revealing that the toxin selectivity at the tissue level in the *Dichotomyctere* freshwater pufferfish is completely different from that in the *Takifugu* marine pufferfish.

**Figure 1:**
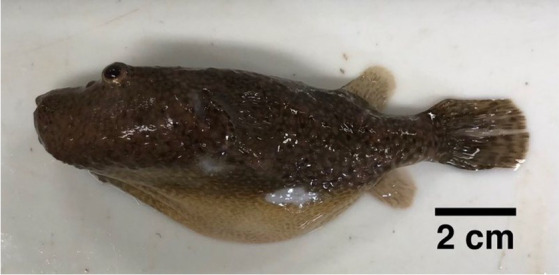
Artificially bred individual of *Pao baileyi*.

The genus *Pao* is the most typical among the pure freshwater pufferfish, comprising approximately 14 species. Most of the species in *Pao* were previously classified under the genera *Tetraodon* and *Monotrete*, primarily living in freshwater and brackish waters of Southeast and South Asia (1). To date, the toxic pufferfish species identified within the genus *Pao* include *Pao fangi* (23), *Pao palembangensis* (45), *Pao suvattii* (46), *Pao leiurus* (47), *Pao turgidus* (36), and *Pao* spp. (24). Artificially bred nontoxic *P. suvattii* exhibited marked selectivity for STXs in oral administration experiments, consistent with the toxin accumulation characteristics of wild *P. suvattii* individuals (34). However, the selective toxin uptake patterns at the tissue level of *Pao* freshwater pufferfish remain unclarified. *Pao baileyi* is a relatively rare freshwater pufferfish distributed in the Mekong River basin spanning Thailand, Laos, and Cambodia, and stands out in the genus *Pao* due to its body being covered in hairlike epidermal appendages, which also lends it ornamental value (48, 49). Nevertheless, the toxicity characteristics of wild *P. baileyi* remain unknown. Therefore, this study investigated the toxicity levels and toxin distribution patterns in *P. baileyi* wild individuals collected from Thailand. Subsequently, *in vivo* intrarectal toxin administration and *in vitro* tissue slice incubation experiments were performed using artificially bred nontoxic *P. baileyi* individuals provided by Osaka Aquarium NIFREL (Figure 1), to explore the kinetic characteristics of STX and toxin uptake profiles at the tissue level in *Pao* freshwater pufferfish. To our knowledge, such a study integrating wild-caught toxicity characteristics, individual-level toxin kinetic properties, and tissue-level toxin uptake profiles is the first of its kind. It provides a robust foundation for studying the molecular mechanisms underlying toxin accumulation and transfer in freshwater pufferfish.

## 2 Materials and methods

### 2.1 Pufferfish specimens

The wild individuals of *P. baileyi* used in this study were collected from Thailand (standard length, 8.2 ± 1.3 cm; body weight, 30.9 ± 11.8 g; *n* = 3) and the artificially bred nontoxic individuals of *P. baileyi* used in both *in vivo* (standard length, 5.1 ± 0.2 cm; body weight, 6.4 ± 0.5 g; *n* = 9) and *in vitro* (standard length, 10.1 cm; body weight, 48.8 g; *n* = 1) experiments were hatched and reared in Osaka Aquarium NIFREL. The three wild individuals were stored intact at −20 °C until dissection and toxin extraction as described in the previous study (27).

### 2.2 Acclimation procedures

The acclimation procedures lasted for seven days, with nine specimens divided equally into three groups, and placed in advance into specialized tanks to acclimate to the water temperature and other experimental conditions. After another one day of acclimation without feeding, the *in vivo* toxin administration experiment was carried out. On the other hand, once the selected larger individual had adjusted to the captive environment, we commenced the *in vitro* tissue slice incubation experiment.

### 2.3 Toxin preparation

TTX (purity > 60%) from the ovaries of *T. rubripes* was prepared, and STX (purity > 80%) from the xanthid crab *Zosimus aeneus* was prepared by solvent partitioning, activated charcoal treatment, and Bio-Gel P-2 (Bio-Rad Laboratories, Inc., Hercules, CA, USA) and Bio-Rex 70 (Bio-Rad) column chromatography according to the previously reported methods (50). Crystalline TTX (Wako Pure Chemical Industries Ltd., Tokyo, Japan) and an aqueous solution of STXs purified from the xanthid crab *Z. aeneus* were used as external standards for the toxin identification and quantification described in Section *2.6. Toxin Qualitative and Quantitative Analyses*.

### 2.4 Intrarectal toxin administration experiment

The intrarectal toxin administration experiment was slightly modified based on the methods described by Zhu et al. (50). The toxin profiles of *P. baileyi* wild individuals investigated in this study indicated that this species possesses potential toxin accumulation capacity, therefore, we believed that appropriately reducing the dose will not only minimize discomfort to the individuals, but also pose no difficulty for quantitative analyses. Then we prepared an STX aqueous solution and administered it to the artificially bred nontoxic individuals using a microliter syringe (Hamilton Company, Reno, NV, USA), achieving a final dose of 5 nmol/fish. Afterwards, these individuals were all quickly returned to their original tanks, and three individuals were randomly picked up at 24, 48, and 72 h to collect intestine, liver, muscle, and skin (including the fins). Each tissue was homogenized in 0.1 M HCl, heated in a boiling water bath for 10 min, and centrifuged at 2300 *g* for 15 min. The supernatant was passed through a FILTSTAR syringe filter (diameter 13 mm, hydrophilic nylon 0.45 μm, Hawach Scientific Co., Ltd., Xi’an City, China), diluted appropriately to exclude matrix effects, and then subjected to toxin identification and quantification.

### 2.5 *In vitro* tissue slice incubation experiment

The tissue slice incubation experiment was essentially performed following the methods described by Zhu et al. (42). In brief, circular tissue slices (8 mm diameter, ~1 mm thick) were prepared from the intestine, liver, muscle, and skin of another *P. baileyi* artificial individual (n = 1), and each slice was incubated with 1.5 mL incubation buffer (160 mM NaCl, 4.8 mM KCl, 23.8 mM NaHCO_3_, 0.96 mM KH_2_PO_4_, 1.5 mM CaCl_2_, 1.2 mM MgSO_4_, 12.5 mM HEPES, and 5.0 mM D-glucose; adjusted to pH 7.4 with NaOH solution) containing TTX (10 nmol/mL) and STX (10 nmol/mL) in a 15 mL plastic tube aerated with Oxygen and Carbon Dioxide at a 9:1 ratio at 20 °C. For the intestine, three slices were randomly sampled from the incubation buffer at 8, 24, 48, and 72 h, respectively. For the liver, muscle, and skin, the same sampling procedure was performed at 24, 48, and 72 h, respectively. Afterwards, tissue slices were weighed after washing with neutral phosphate buffer (0.15 M NaCl and 0.01 M Na_2_HPO_4_; adjusted to pH 7.0 with 0.15 M NaCl and 0.01 M NaH_2_PO_4_), and then 1 mL of 0.1% acetic acid was added to each slice, and the slices were disrupted by ultrasonication and heated in a aluminum block bath (Dry Thermo Unit, TAITEC Corp., Saitama, Japan) for 10 min. After centrifugation at 20000 *g* for 20 min, the supernatant was passed through a FILTSTAR syringe filter (diameter 13 mm, hydrophilic nylon 0.45 μm, Hawach Scientific), diluted appropriately to exclude matrix effects, and then submitted to toxin identification and quantification as mentioned below.

### 2.6 Toxin qualitative and quantitative analyses

Toxins were analyzed by liquid chromatography with tandem mass spectrometry (LC–MS/MS) and high-performance liquid chromatography with post-column fluorescence derivatization (HPLC-FLD), respectively.

For TTX identification and quantification, an ACQUITY UPLC H-Class System comprised of a Quaternary Solvent Manager, a Sample Manager (Waters Corp., Milford, MA, USA), and an XBridge® BEH Amide column XP (2.1 × 150 mm, particle size 2.5 μm, Waters Corp.), was used for the chromatography. The column was held at 60 °C and the Sample Manager was set to 10 °C. Mobile phases were as follows; B: 0.1% formic acid in 20 mM ammonium formate (water/formic acid/ammonium formate = 490:0.5:10, *v/v*); C: 100% acetonitrile. The gradient conditions and flow rate are summarized in Table 1. The eluate was analyzed using an Xevo™ TQ-S micro mass spectrometer (Waters Corp.) operating in positive electrospray ionization mode. Ionization parameters were set as follows: desolvation temperature at 600 °C, source block temperature at 150 °C, and cone voltage at 35 V. Qualitative and quantitative detection of TTX was performed via multiple reaction monitoring (MRM) at *m/z* 302 and 162, respectively, with *m/z* 320 designated as the precursor ion under a collision energy of 20 V, utilizing the MassLynx™ NT operating system (Waters Corp.).

**Table 1:** Gradient program in LC–MS/MS analyses.

Time (min)	Flow rate (mL/min)	% B	% C	Curve
Initial	0.4	16	84	Initial
3	0.4	35	65	6
8	0.4	50	50	6
10	0.4	16	84	1

For analyses of STXs, chromatographic separation was performed using Prominence Ultra-Fast Liquid Chromatography (Shimadzu Corp., Kyoto, Japan) with a LiChroCART® Superspher® 100 RP-18(e) column (4.0 × 250 mm, particle size 4 μm, Kanto Chemical Co., Inc.). The separation of STXs was achieved using a mobile phase containing 2 mM 1-Heptanesulfonic acid sodium salt in 4% acetonitrile-30 mM ammonium phosphate buffer (pH 7.3), delivered at a flow rate of 0.8 mL/min. The column was maintained at 35 °C. The eluate from the column was mixed continuously with 50 mM periodic acid and 0.2 M KOH containing 1 M ammonium formate and 50% formamide, and heated at 65 °C. Fluorophore formation was monitored at 392 nm with 336-nm excitation through an RF-20A XS Prominence Fluorescence Detector (Shimadzu Corp.).

The limit of detection (LOD) and limit of quantification (LOQ) for TTX were 0.009 nmol/mL (*S/N* = 3) and 0.03 nmol/mL (*S/N* = 10), for STX were 0.012 nmol/mL (*S/N* = 3) and 0.04 nmol/mL (*S/N* = 10). The spike-and-recovery test showed that the STX recovery was > 90%.

### 2.7 Statistical analyses

Student’s t-test was performed using GraphPad Prism version 10.3.1 for Windows (GraphPad Software, Inc., Boston, MA, USA), a *p*-value of less than 0.05 was considered statistically significant.

## 3 Results

**Figure 2:**
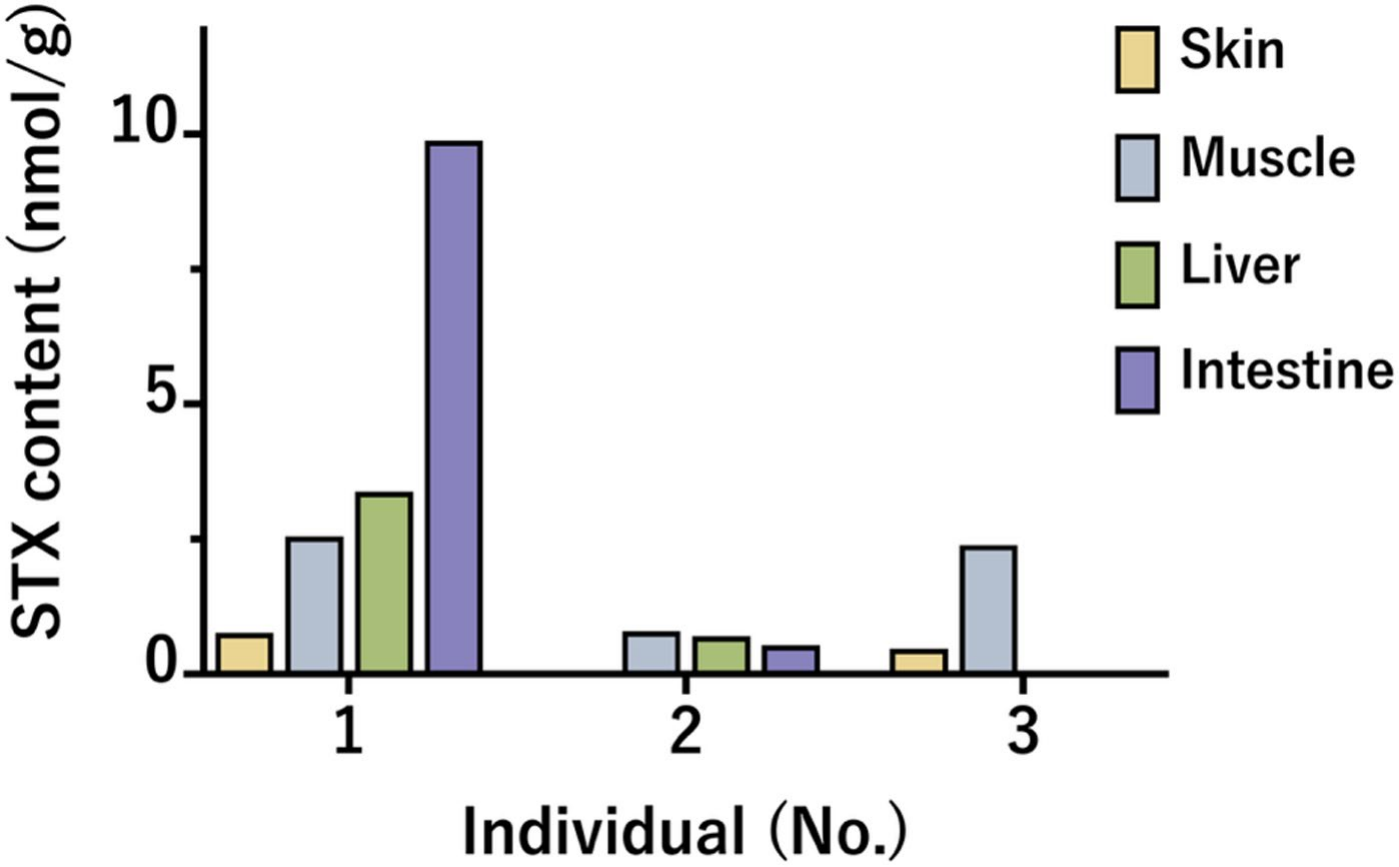
STX content in the skin, muscle, liver, and intestine of *P. baileyi* wild individuals collected from Thailand.

Like other *Pao* freshwater pufferfish reported to date, no toxins other than STXs (including TTX, GTXs, and C toxins) have been detected in *P. baileyi* wild individuals. STX was the only toxic component in each tissue (see the chromatograms of Supplementary Figures S1, S2). Macroscopically, significant individual variation was observed (Figure 2). Wild individual No. 1 exhibited higher STX concentrations in all tissues (0.76–9.88 nmol/g), whereas No. 2 and No. 3 contained lower STX levels, ranging from 0–0.79 nmol/g and 0–2.39 nmol/g, respectively. The skin showed the lowest toxicity among all tissues, with STX concentrations of 0.76 nmol/g (No. 1), 0 (not detected, No. 2), and 0.47 nmol/g (No. 3). No. 3 showed no detectable STX in either the liver or intestine, and No. 1 and No. 2 contained only 3.38 and 0.7 nmol/g of STX in the liver, respectively. On the other hand, the intestine of No. 1 possessed the highest STX concentration (9.88 nmol/g). Muscle was the only site where STX was detected in all three individuals, with concentrations of 2.55 nmol/g, 0.79 nmol/g, and 2.39 nmol/g, respectively.

**Figure 3:**
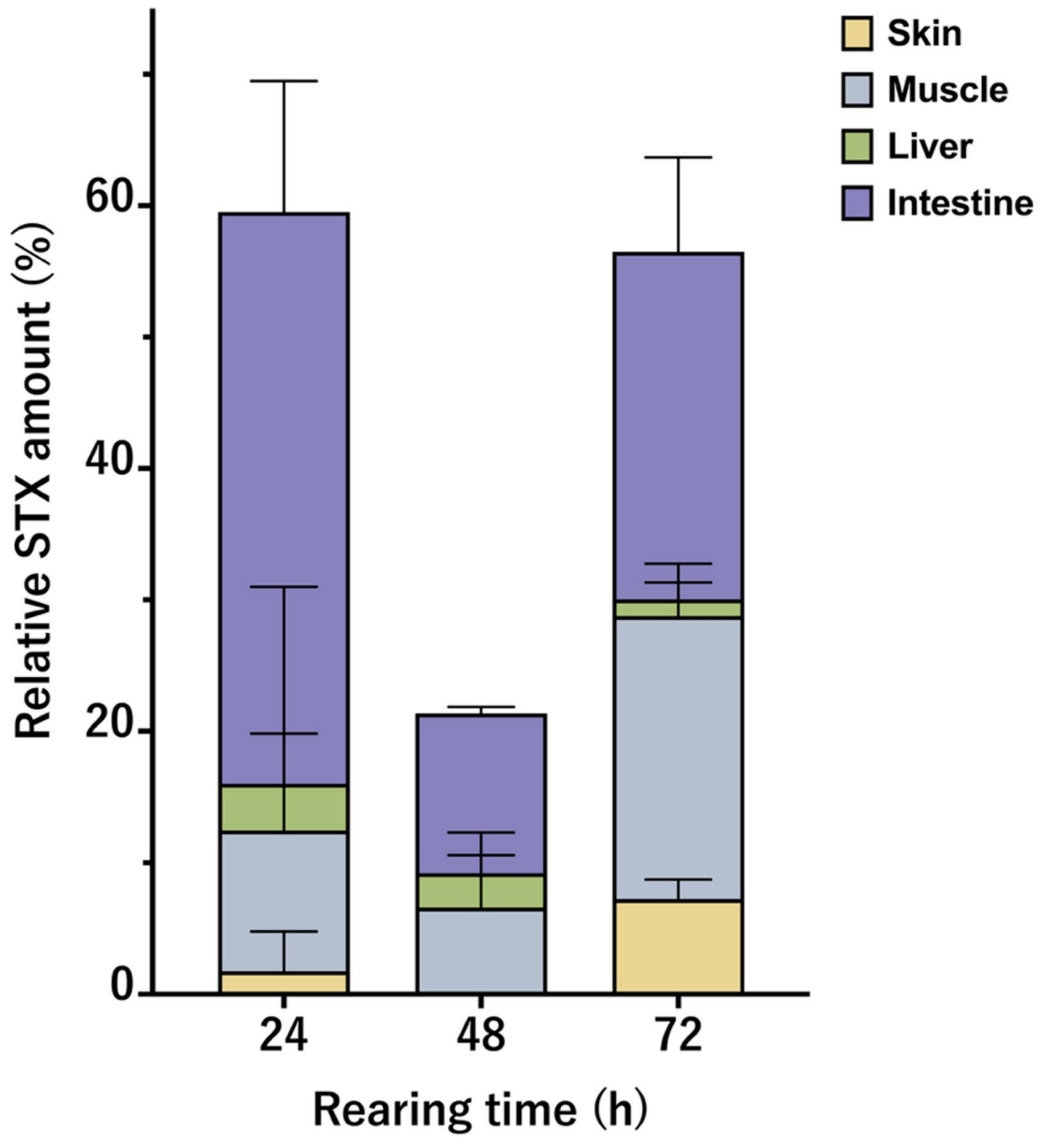
Relative STX amount (molar %) in the tissues (skin, muscle, liver, and intestine) of artificially bred individuals of *P. baileyi* at 24, 48, and 72 h after being administered STX intrarectally. Data are shown as means (columns) and standard deviations (SDs) (error bars) of three individuals.

Figure 3 shows the results of intrarectal toxin administration using artificially bred nontoxic individuals (*n* = 9). Overall, the levels of STX entering *P. baileyi* via intrarectal administration and accumulating in the skin, muscle, liver, and intestine exhibited an upward-downward-upward trend. The sum of relative STX amount in all tissues at 24, 48, and 72 h was 59.52 ± 35.34%, 21.37 ± 7.58%, and 56.49 ± 13.94%, respectively. At 24 h post-administration, the intestine, as the site of prolonged direct contact with the toxin, accumulated 43.51% (average value) of STX, subsequently decreasing to an average of 12.16% at 48 h, and finally rising to 26.45% (average value) at 72 h. The skin accumulated 1.75% STX within the initial 24 h, but no detectable STX was present at 48 h. After another 24 h, 7.24% of STX was ultimately transferred to the skin. The relative STX amount in the muscle remained stable during the first 48 h, with 10.7% at 24 h and 6.59% at 48 h, before increasing significantly to 21.53% at 72 h. Although the liver exhibited a stable relative amount of STX similar to muscle during the early to mid-experimental period (3.56% at 24 h and 2.62% at 48 h), the liver showed a slight decrease in the relative STX amount at 72 h, reaching 1.27%.

**Figure 4:**
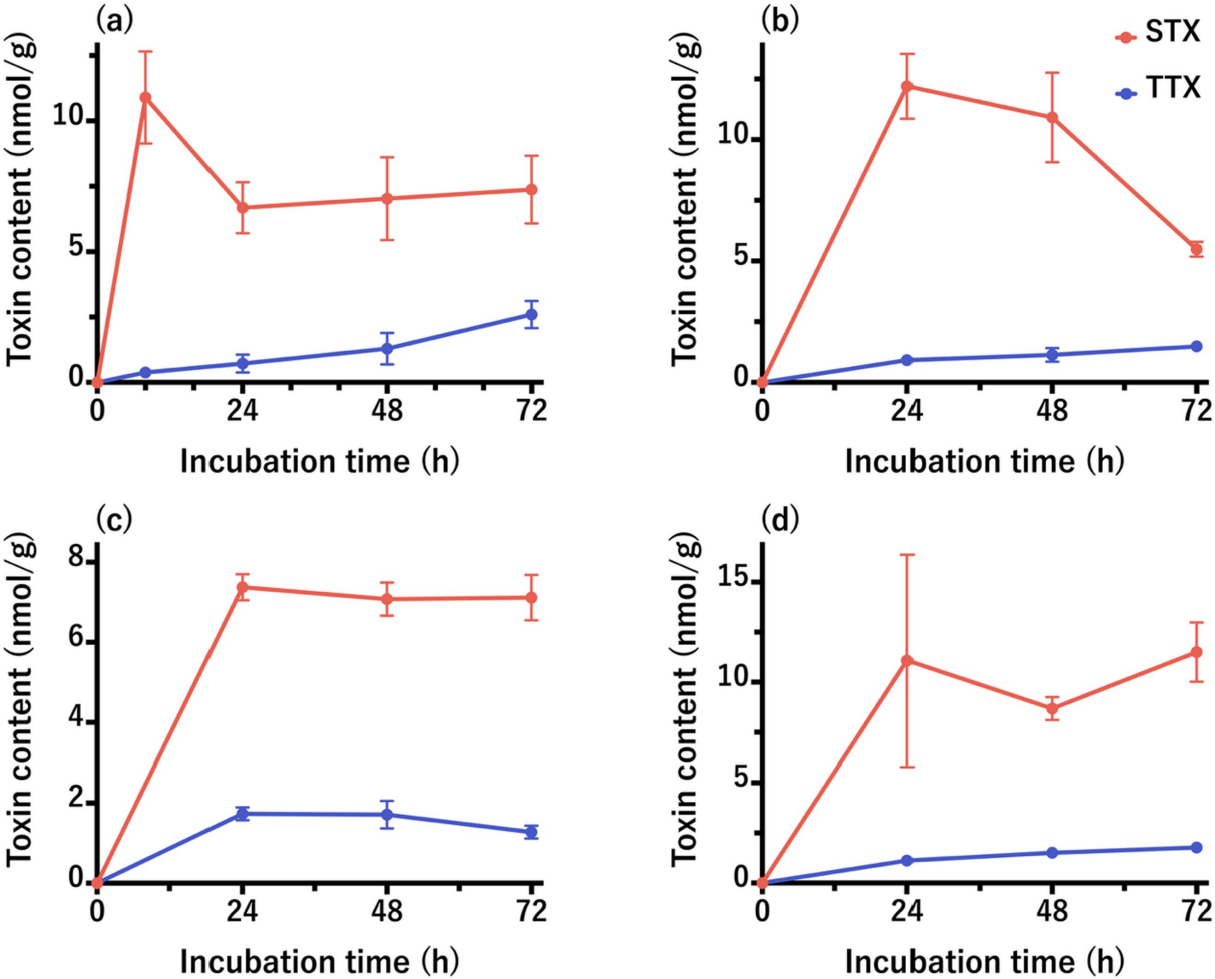
Changes in toxin content in the intestine **(a)**, liver **(b)**, muscle **(c)**, and skin **(d)** slices of *P. baileyi* during incubation for up to 72 h. Each slice was incubated with buffer containing TTX (10 nmol/mL) and STX (10 nmol/mL). Data are shown as means (dots) and SDs (error bars) of three slices.

The results of the *in vitro* tissue slice incubation are presented in Figure 4. Tissue slices from the intestine, liver, muscle, and skin of artificially bred nontoxic *P. baileyi* were prepared and placed in a solution containing TTX and STX. The slices were incubated continuously for 72 h, and the content of toxins taken up by each slice over time was analyzed and quantified. Tissue slices of intestine reached the peak uptake of STX at 8 h, averaging 10.9 nmol/g, then dropped sharply to 6.68 nmol/g by 24 h (Figure 4a). The uptake levels of STX remained above 7 nmol/g thereafter (averaging 7.03 nmol/g at 48 h and 7.37 nmol/g at 72 h). For TTX taken up by the intestine, although contents steadily increased from 0.38 nmol/g at 8 h to 2.59 nmol/g at 72 h, they remained remarkably lower overall than STX contents (*p* < 0.05). In contrast to the intestine, tissue slices of liver showed the highest content of STX taken up at 24 h (12.2 nmol/g), followed by a steady decline until 72 h (5.49 nmol/g) (Figure 4b). On the other hand, TTX content in the liver slices was low at 24 h (0.92 nmol/g) and remained stable through 72 h (1.49 nmol/g). The muscle slices also exhibited the highest uptake of STX over 24 h (7.38 nmol/g), however, unlike the declining trend observed in the intestine and liver slices after reaching their first peak, the muscle slices maintained similar STX uptake capacity at 48 and 72 h after reaching their maximum STX content, with average values of 7.08 nmol/g and 7.12 nmol/g, respectively (Figure 4c). On the other side, average content of TTX dropped to 1.28 nmol/g at 72 h after stable passing through 24 h (1.74 nmol/g) and 48 h (1.71 nmol/g), all *p*-values were less than 0.05. The STX content curve in the skin slices over time exhibited striking one snag after another: it first reached a local peak at 24 h (11.08 nmol/g), then decreased to 8.68 nmol/g at 48 h, before rising again to achieve the maximum STX content (11.5 nmol/g) at 72 h (Figure 4d). The content curve of TTX taken up by tissue slices of skin resembled that by the liver slices, rising slowly to 1.12 nmol/g within 24 h, then plateauing after reaching 1.51 nmol/g at 48 h, and finally showing an average uptake of 1.78 nmol/g at 72 h, which was clearly different from the STX content at the same time point (*p* < 0.05).

## 4 Discussion

The present study investigated the toxin profiles of wild *P. baileyi*, revealing that this species is a *Pao* freshwater pufferfish exhibiting high toxicity in the intestine and muscle, but relatively weak toxicity in the liver and skin. The *in vivo* intrarectal toxin administration using artificially bred nontoxic *P. baileyi* individuals revealed the kinetic transfer pathway of STX: STX enters the body via the intestine, undergoes a brief retention in the liver, and then is transferred to the muscle/skin. In addition, tissue slices from artificially bred nontoxic *P. baileyi* individuals were prepared for *in vitro* incubation, which confirmed the selective uptake of STX by *Pao* freshwater pufferfish at the tissue level for the first time. Meantime, it also demonstrated the changes in toxin uptake ability of various tissues *in vitro* over time, which to some extent supported and corroborated the results from the investigation of toxicity profiles of wild *P. baileyi* and from *in vivo* intrarectal administration. Due to sampling constraints, only three wild *P. baileyi* were available. Furthermore, to ensure the results of *in vitro* tissue slice incubation would not be affected by individual variations, all tissue sections were taken from the same individual. Thus, the sample size was limited to a certain extent, however, given that similar results were also observed in our preliminary experiments, the results of our study are generalizable, which comprehensively elucidate the toxicological profiles of representative *Pao* freshwater pufferfish.

This study investigated the types and distribution profiles of toxins present in *P. baileyi* wild individuals through instrumental analyses and found that the skin and liver are containing relatively low concentration of STX. The liver of freshwater pufferfish is believed to be almost nontoxic or low toxic, while normally the skin is the most toxic part of freshwater pufferfish (24, 27, 33, 36, 45). The accumulation of toxins in the skin has ecological significance: many pufferfish are found to have secretory glands or secretory cells in the skin (51–53) and release toxins from the skin to defend against enemies when exposed to external stimuli (54, 55). Gao et al. (56) evaluated the TTX uptake ability of *T. rubripes* tissues and found that the TTX uptake ability of the skin differs significantly between juvenile and adult pufferfish with different basal cell properties in the skin through immunohistochemical observation. All *P. baileyi* wild individuals in this study were immature juveniles, thus, there exists the possibility that their skin exhibits toxin profiles markedly different from those of adult freshwater pufferfish, due to the underdevelopment of structures such as secretory cells or basal cells. Comparative studies including immunohistochemical experiments on juvenile and adult freshwater pufferfish require further research. Among the three wild individuals in this study, considerable STX was detected in the intestine of both No. 1 and No. 2, but not in that of No. 3. Notably, the intestine of No. 1 contained a far greater STX concentration than the other tissues. STX is generally considered an exogenous toxin, so its abundance in the food chain could be easily reflected in the STXs amount in the intestine, and it also directly determines STX level in pufferfish. Zhu et al. (27) investigated the intra-body distribution of STXs in a freshwater pufferfish of the genus *Pao* collected from two regions and found that habitat is a critical factor influencing STXs level in freshwater pufferfish, as varying nutrient contents in different environments support distinct STX-producing cyanobacteria populations, consequently, freshwater pufferfish accumulate differing STXs amounts through their diet. Therefore, although all three wild individuals used in this study were collected from Thailand, it is considered that the habitats of No. 1 and No. 2 should differ greatly from that of No. 3, and the abundance of STX in their respective food chains is also likely to be vastly different. The investigation and identification of toxic organisms within the stomach contents of freshwater pufferfish, along with the tracing to the source of STXs, should be the focus of subsequent research.

It was revealed through intrarectal administration that STX undergoes the following kinetics in *Pao* freshwater pufferfish: intestine → liver → muscle/skin. The prevailing view holds that STXs are exogenous toxins primarily originating from the food chain. As the initial point of interaction with food, the intestine is most susceptible to absorbing STXs from the diet, serving as the primary entry point of STXs into the body. Consequently, at the initial 24 h of intrarectal STX administration, the highest proportion of STX accumulates in the intestine. Zhu et al. (50) conducted an intrarectal TTX/STX administration experiment using a euryhaline marine pufferfish and found that the relative STX amount in the intestine peaked at 24 h and then declined greatly by 48 h, consistent with the STX kinetics in the same tissue observed in the current study. Although the STXs concentration in the liver of wild individuals has been shown relatively low, as a tissue responsible for detoxification and metabolism, the liver seemed to function as a transportation hub from 24 h to 72 h: transporting STX primarily distributed in the intestine at 24 h to other tissues, ultimately resulting in a state where STX is evenly distributed in the intestine, muscle, and skin by 72 h. Similar functions have also been observed in the liver of marine pufferfish (57), though the destinations for TTX transferred from the liver are the skin and ovaries, excluding the muscle. However, the average relative STX amount accumulated in the muscle over 72 h in this study is 21.53%, far exceeding the 7.24% of STX in the skin during the same period. Laobhripatr et al. (58) investigated the toxicity of the freshwater pufferfish *P. fangi* from Thailand and revealed that toxicity in the muscle ranked next to in the skin and the gonads, reaching a maximum of 457 MU/g (equivalent to 184 nmol/g STX). Food poisoning incidents caused by accidentally consuming toxic freshwater pufferfish have become increasingly common in Thailand in recent years (59), raising urgent concerns about food safety and societal issues. Combined with the findings of this study, it indicates that wild *Pao* freshwater pufferfish inhabiting Thailand possess the potential to transfer and accumulate STXs in the muscle. Therefore, we urge residents to refrain from consuming wild freshwater pufferfish under any circumstances, and hope that relevant administrative departments would intensify public awareness campaigns regarding food safety.

There are generally three types of *in vivo* toxin administration for pufferfish: oral gavage, intramuscular injection, and intrarectal administration. Gao et al. (34) administered feed homogenate containing STX to artificially nontoxic *P. suvattii* by oral gavage and found that the relative proportion of STXs accumulated in tissues of *P. suvattii* within 48 h was no more than 40%, however, in this intrarectal toxin administration experiment using *P. baileyi* individuals, the amount of STX accumulated in the tissues exceeded 50% of the administered STX amount at both 24 h and 72 h, showing that intrarectal administration is more effective than oral gavage in terms of toxin accumulation. In their study, the amount of STX accumulated in the intestine and skin showed a decreasing trend over a period of 24 h to 48 h, regardless of sex. Similarly, STX accumulated in the intestine and skin of *P. baileyi* individuals also decreased from 24 h to 48 h, indicating that both intrarectal administration and oral gavage could reflect the initial kinetic characteristics of STX after being absorbed through the intestine and entering the body: within the first 48 h, STX concentrated from peripheral tissues toward visceral tissues. On the other hand, Ngy et al. (36) intramuscularly administered STXs into artificially nontoxic *P. turgidus* and found that STXs were transferred via the blood from muscle into other tissues, especially the skin. The STXs amount in the skin was observed highest among the tissues starting 12 h after the injection and persisted for up to 48 h. However, in contrast to the persistent increase in STXs accumulated in the skin of *P. turgidus*, the amount of STXs in the skin of *P. baileyi* underwent a dynamic change over 72 h: first a slight increase, followed by a decrease, and then a significant increase. The primary explanation for this difference between the two types of administration is presumed to be the injection site: muscle and skin are linked together. Shiomi (60) found that during the slow defrosting of marine pufferfish *T. rubripes*, TTX migrated from toxic skin to nontoxic muscle, suggesting that due to the close connection between muscle and skin, toxins tend to migrate from high-concentration sites to low-concentration sites. Therefore, the proximity of muscle to skin allows STXs entering the body via intramuscular injection to be more readily transferred to and retained by the skin. However, given that intramuscular injection inadvertently accelerates the accumulation of toxins in the skin, failing to fully characterize the toxin’s kinetics within the body, and it may cause pain and trigger a stress response in pufferfish, intrarectal administration is believed to be more appropriate.

To our knowledge, this is the first systematic study integrating the toxin profiles of wild individuals, individual-level toxin kinetic features, and tissue-level toxin uptake characteristics in freshwater pufferfish. The current study is also the first to elucidate TTX/STX selective uptake characteristics of the *Pao* freshwater pufferfish tissues. In the investigation on TTX/STXs selectivity in the intestine, liver, and skin of euryhaline freshwater pufferfish *D. fluviatilis*, STXs taken up by three tissues at almost all time points did not exceed 10 nmol/g (42). Compared with *D. fluviatilis*, it is found that the tissues of *P. baileyi* have a stronger overall ability to take up STX. Regarding the dynamic changes of STX within tissues over the 24-to 72 -h period, the liver and skin in the *in vitro* tissue slice incubation experiment exhibited trends consistent with those observed in the *in vivo* intrarectal administration experiment: STX taken up by the liver showed a decreasing trend starting at 24 h and persisting through 72 h; for skin, STX decreased from 24 h to 48 h and then increased from 48 h to 72 h. However, STX taken up by the intestine reached a maximum at 8 h, decreased before 24 h, and finally remained stable from 24 h to 72 h, which is totally different from the observation of STX kinetics in the intestine in intrarectal administration. Gao et al. (56) conducted a similar tissue slice incubation experiment using intestine of *T. rubripes* and supposed that TTX was taken up from the epithelial cells of the villi into the lamina propria, and gradually transferred to the muscle layer. Nevertheless, they emphasized that it is unclear how such uptake of toxin by the intestine slices is involved in *in vivo* intestinal toxin absorption. The underlying explanation for this discrepancy requires further investigation. Since we found that the muscle of *P. baileyi* wild individuals contained considerable STX, and the muscle was also identified as an important destination for STX transfer within the body during intrarectal administration, we performed *in vitro* tissue slice incubation on muscle for the first time. Despite exhibiting slightly lower STX uptake capacity compared to the other three tissues, muscle still took up over 7 nmol/g of STX at its peak. Considering that muscle weight contributes significantly to the total body weight of the pufferfish, it is believed that all tissues of *P. baileyi*, including the muscle, are unsuitable for consumption.

In the current tissue slice incubation experiment, a 1.5 mL toxic solution containing TTX and STX (15 nmol/tube, respectively) was used, however, the concentration of STX taken up by each tissue was significantly higher than that of TTX, demonstrating the tissue-level selective uptake of STX by freshwater pufferfish *P. baileyi*. Some *in vitro* tissue slice incubation experiments have evaluated the selective uptake characteristics of TTX and STX in pufferfish from various genera within the family Tetraodontidae, such as the liver, intestine and skin of *T. rubripes* selectively took up TTX (42, 61), while the intestine, liver, and skin of *D. fluviatilis* selectively took up STX (42). Numerous studies have demonstrated that *Pao* freshwater pufferfish possess only STXs rather than TTX, however, the tissue-specific selectivity of TTX/STX within the genus *Pao* remained unclear until this study revealed that tissues of freshwater pufferfish belonging to the genus *Pao*, like that belonging to the genus *Dichotomyctere*, could selectively take up STX. Zhu et al. (24) conducted simultaneous genetic-based phylogenetic and toxin analyses using freshwater pufferfish collected from Cambodia and attributed the freshwater pufferfish preference for STX to their possession of the tributyltin-binding protein type 2 (TBT-bp2) gene but lack of the pufferfish STX-and TTX-binding protein (PSTBP) gene. PSTBP is a soluble glycoprotein isolated from *Takifugu pardalis* plasma, showing the binding capacity for TTX (62). On the other hand, TBT-bp2 is a homologue of α1-acid glycoprotein isolated from the blood of Japanese flounder, which has been found to share a similar structure with PSTBP and is presumed to be the evolutionary origin of PSTBP (63, 64). In the study of Zhu et al. (24) mentioned above, it is found that differences in the TBT-bp2 sequences result in varying STXs concentrations in *Pao* freshwater pufferfish. In this study, the intestine, liver, muscle, and skin showed an extremely strong preference for STX and a commendable uptake ability of STX, however, during the tissue slice incubation, the critical role played by TBT-bp2 in the selective uptake of STX by the tissues, the changes in TBT-bp2 expression levels, and whether any unknown proteins may form synergistic interactions with TBT-bp2, all need thorough research as one direction for future work.

## 5 Conclusion

This study identified *P. baileyi* as a freshwater pufferfish containing only STX and exhibiting high STX concentration in both intestine and muscle. Meantime, intrarectal toxin administration reveals the kinetic transfer pathway of STX, which is absorbed through the intestine into the body, undergoes a brief retention in the liver, and is subsequently transferred to the muscle/skin. Both toxicity investigation on wild individuals and intrarectal toxin administration demonstrate that the muscle of *P. baileyi* possesses a remarkable capacity for STX accumulation, a trait that is exceptionally rare among freshwater pufferfish. Additionally, through tissue slice incubation experiments, we discovered that the *Pao* freshwater pufferfish selectively take up STX at the tissue level. The dynamic trends observed in tissues such as liver and skin largely corresponded with the results from intrarectal toxin administration, thereby partially validating the reliability of this multilevel study. Notably, in the intrarectal toxin administration, although STX showed a tendency to migrate from peripheral tissues toward visceral tissues within the initial 48 h, the overall relative STX content at 48 h decreased significantly compared to 24 h, and then it rebounded substantially at 72 h. It is speculated that a portion of STX may have transferred to certain tissues beyond the intestine, liver, muscle, and skin, therefore, their dynamic changes could not be tracked. In the future, exploring other potential STX-accumulating tissues will be considered. Such a comprehensive elucidation of the toxin possession, accumulation, and uptake profiles in *P. baileyi* will not only enhance our understanding of toxin accumulation behavior and metabolic processes in *Pao* freshwater pufferfish, but also provide a theoretical foundation for investigating the molecular mechanisms underlying toxin accumulation in freshwater pufferfish.

## Data Availability

The raw data supporting the conclusions of this article will be made available by the authors, without undue reservation.
