# Supplementary material for: A comprehensive study on freshwater pufferfish Pao baileyi: profiles of toxin possession, accumulation, and uptake
Source: Front Vet Sci. 2026 Jul 27;13:1899689. doi: 10.3389/fvets.2026.1899689 (PMC13454042; doi:10.3389/fvets.2026.1899689)
Supplement: Supplementary file 1 [file Data_Sheet_1.PDF]

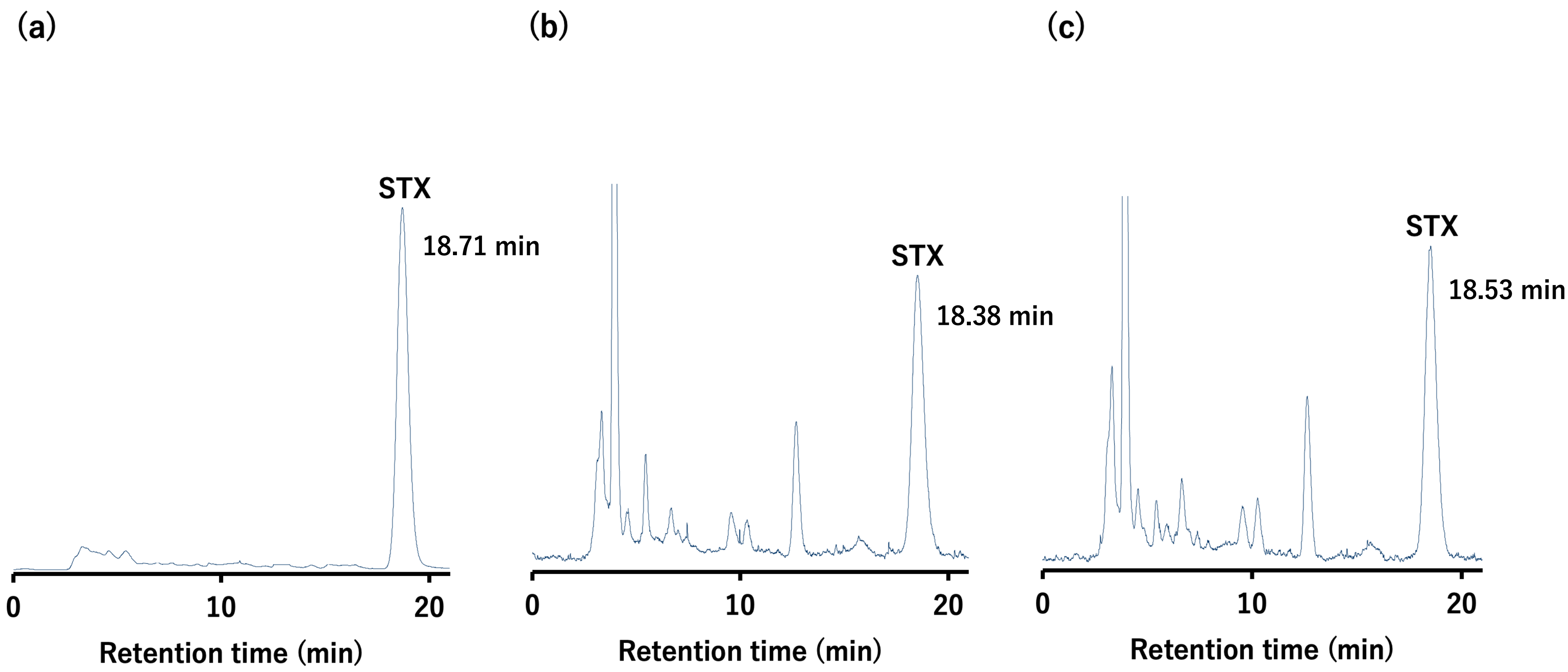


**Fig. S1.** HPLC-FLD chromatograms of STX standard (a), the liver extract of wild individual No. 1 (b), and muscle extract of wild individual No. 3 (c). The concentration of STX standard is 4 nmol/mL. The injection volume is 10 µL.

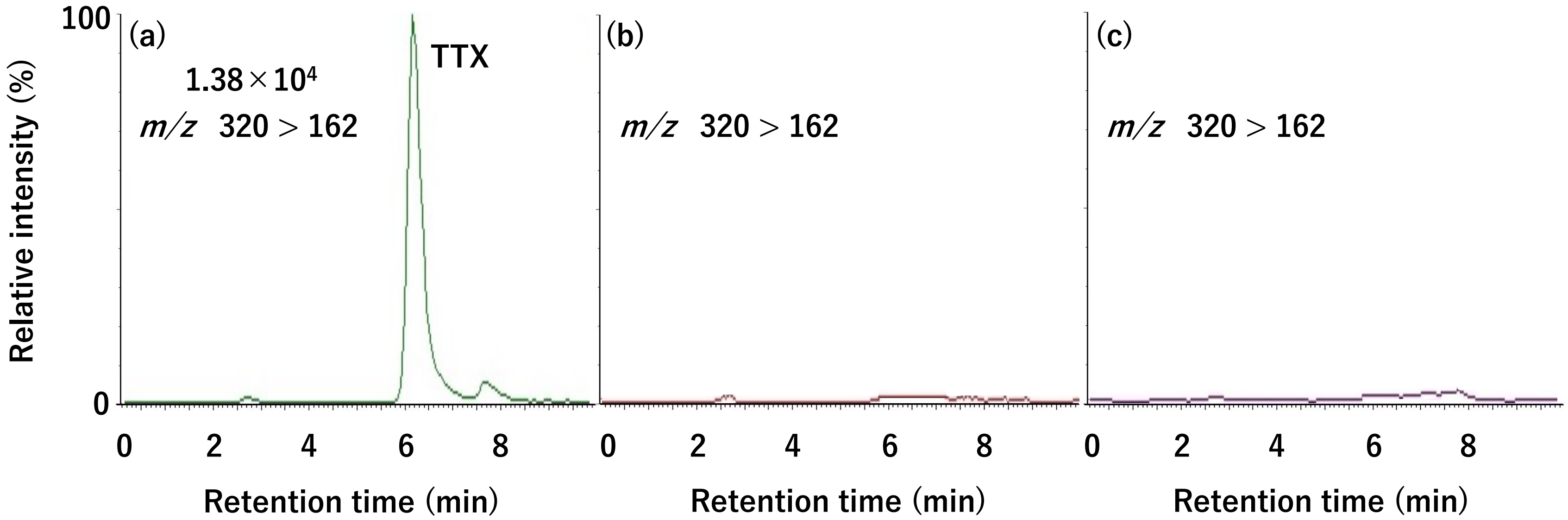


**Fig. S2.** LC-MS/MS chromatograms of TTX standard (a), the intestine extract of wild individual No. 1 (b), and muscle extract of wild individual No. 3 (c). The concentration of TTX standard is 0.689 nmol/mL.
